# Benefit–Cost Analysis for Earthquake-Resilient Building Design and Retrofit: State of the Art and Future Research Needs

**DOI:** 10.1061/nhrefo.nheng-1910

**Published:** 2024

**Authors:** Yating Zhang, Juan F. Fung, Dustin Cook, Katherine J. Johnson, Siamak Sattar

**Affiliations:** 1 Dept. of Civil and Environmental Engineering, Univ. of Maryland, College Park, MD 20742; PREP Researcher, Earthquake Engineering Group, National Institute of Standards and Technology, Gaithersburg, MD 20899.; 2 Economist, Applied Economics Office, National Institute of Standards and Technology, Gaithersburg, MD 20899; 3 Earthquake Engineering Group, National Institute of Standards and Technology, Gaithersburg, MD 20899.; 4 Earthquake Engineering Group, National Institute of Standards and Technology, Gaithersburg, MD 20899.; 5 Earthquake Engineering Group, National Institute of Standards and Technology, Gaithersburg, MD 20899.

**Keywords:** Benefit–cost analysis (BCA), Earthquake risk reduction, Building codes, Seismic retrofit, Performance objectives

## Abstract

This paper reviews the state of the art in using benefit–cost analysis (BCA) to inform earthquake risk reduction decisions by building owners and policymakers. The goal is to provide a roadmap for the application and future development of BCA methods and tools for earthquake risk reduction. Our review covers three earthquake risk reduction measures: adopting up-to-date building codes for new construction, designing new buildings to exceed code requirements, and retrofitting deficient existing buildings. We highlight the factors that influence the cost-effectiveness of building design and retrofit, as well as tactics for increasing the cost-effectiveness of risk reduction strategies. We also present BCA results, methods, and data sources used in the literature to help researchers and practitioners design and conduct a reliable and robust BCA study. In the process, we develop a set of opportunities and challenges for applying BCA to new areas of research, as well as key gaps and limitations in current BCA approaches, including further investigation of above-code design, incorporation of code implementation and enforcement into BCA, quantification of environmental benefits of seismic retrofits, and optimization of seismic retrofits with energy upgrades. Overall, our review provides practical guidance and useful insights into BCA with the goal of increasing the earthquake resilience and economic efficiency of buildings in the United States.

## Introduction

**Fig. 1.**
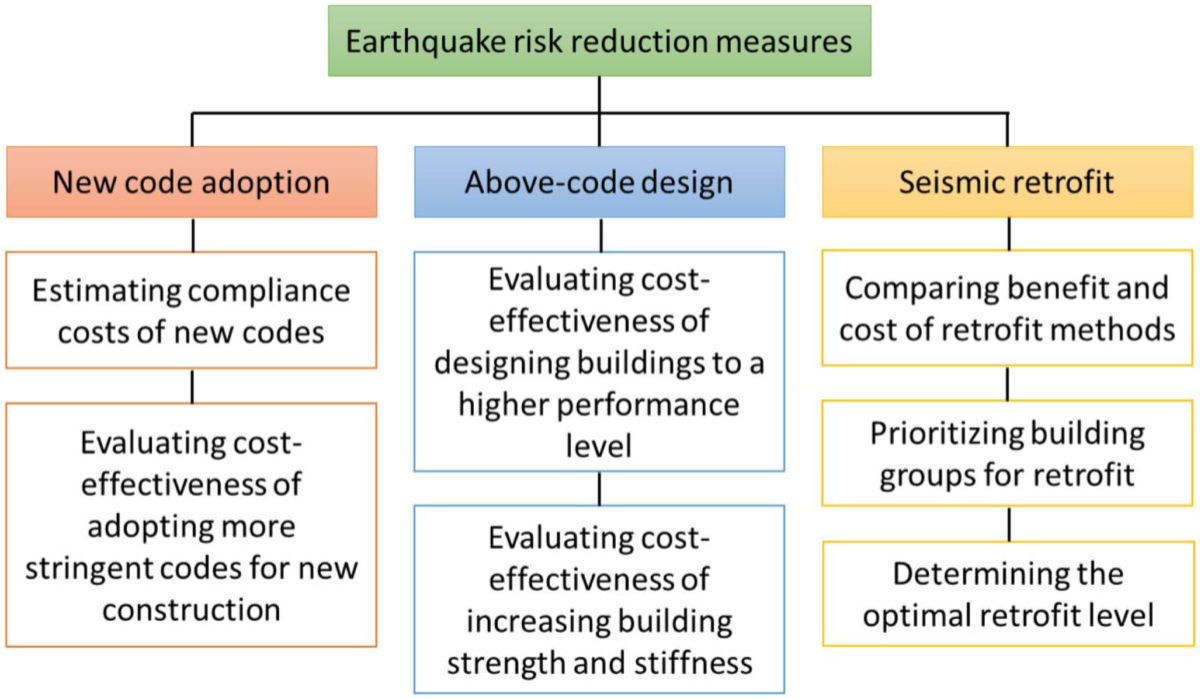
Use of benefit–cost analysis in earthquake mitigation studies. White boxes enumerate example applications.

Benefit–cost analysis (BCA) is widely used in the engineering decision-making process for risk reduction. It evaluates future risk reduction benefits and compares the benefits to the investment costs. When the total benefit is greater than the total cost, the investment is considered cost-effective (FEMA 2009; Fung et al. 2022b). The evaluation criteria can be adjusted based on project needs and local policy requirements. In earthquake preparedness and mitigation practices, BCA has been utilized to determine the cost-effectiveness of adopting up-to-date building codes, designing buildings to exceed code requirements, and retrofitting deficient existing buildings, as illustrated in Fig. 1.

Building codes that reflect up-to-date construction methods and technologies can improve life safety and protect buildings from the effects of natural hazards (ICC 2022; FEMA 2020c). However, new codes can also lead to increased design, construction, and inspection costs, which may prevent state and local governments from implementing more stringent requirements (NEEP 2021; FEMA 1998). A recent study by the Federal Emergency Management Agency (FEMA 2020b) found that 65% of counties, cities, and towns in the United States have not adopted a modern building code [the 2015 and 2018 editions of the international codes (I-Codes)]. Compliance costs and mitigation savings are important considerations for code adoption (FEMA 2020a, b). Previous studies have examined whether compliance with new codes substantially increases construction costs compared to adherence to old codes (NAHB 2018), and whether the benefits of new codes outweigh the costs (NIBS 2019). These studies suggest that the value of adopting new codes in highly seismic regions is undisputed. However, there is a long-standing debate about the cost-effectiveness in regions with moderate seismic risk (Nikellis et al. 2019; Joyner and Sasani 2018; NEHRP 2013; Nordenson and Bell 2000).

Another area of research is the use of BCA to support above-code design. In the US, life safety represents the minimum code requirements that allow buildings to sustain extensive damage after an earthquake, as long as the buildings retain sufficient capacity to withstand aftershocks, and their nonstructural components do not pose a life-threatening hazard (ASCE 2017). However, in highly seismic regions, building codes cannot prevent costly repairs or loss of building functions and services after an earthquake (NIST 2021; Porter 2021; Sattar et al. 2018). This calls for above-code design to achieve higher performance objectives, such as functional recovery or immediate occupancy (Cook and Sattar 2022; Porter 2021; NIBS 2019; Kutanis and Boru 2014). Immediate occupancy means that a building remains safe for occupancy after an earthquake. Specifically, the structure retains its pre-earthquake strength and stiffness, and building access and life safety systems remain operational, but other nonstructural components may not function immediately (ASCE 2017). Functional recovery, which is under active development, is defined as “a post-earthquake performance state in which a building is maintained, or restored, to safely and adequately support the basic intended functions associated with its pre-earthquake use or occupancy” (NIST 2021). The key question addressed in the literature is whether designing buildings to exceed code requirements provides greater net benefits than conforming to existing codes (Fung et al. 2022a; NIBS 2019; Kutanis and Boru 2014; Porter et al. 2006). One of the themes that emerges from our review is that there are many gaps and research opportunities for the application of BCA to support investments in functional recovery design.

Furthermore, older buildings are more susceptible to earthquake damage due to structural deficiencies and deterioration (ATC 2010b). There is an extensive literature assessing the value of seismic retrofits in reducing casualties and building losses over the remaining life of the building or in the event of an unforeseeable large earthquake. The literature addresses questions such as: Is seismic retrofit more economical than demolition and replacement? Do currently available technologies allow older buildings to attain desired performance improvements at acceptable cost? Which strengthening method is most effective in terms of building performance and retrofit costs? Answering these questions helps inform policymaking and resilience planning for earthquake-prone communities (Paxton et al. 2017; Goettel 2016; Gibson et al. 2014). Another branch of research investigates the optimal level of retrofitting, either by minimizing life-cycle costs (e.g., Vitiello et al. 2017; Kappos and Dimitrakopoulos 2008) or by maximizing net present value (e.g., Galanis et al. 2018). The optimal level of retrofitting can be used to guide the design of cost-effective retrofits.

The objectives of this study are to (1) review the literature on BCA for building design and retrofits targeting different levels of seismic performance; (2) identify the factors that influence the cost-effectiveness of building design and retrofits; and (3) explore the opportunities and challenges of using BCA to support decision-making for earthquake-resilient buildings. To enhance the comprehensiveness of this review, we also include studies that delve into benefit analysis, cost estimation, or loss prediction, which are important components of BCA. Researchers may examine benefits or costs independently when significant uncertainties are associated with cost or benefit quantification (e.g., business interruption, community resilience, greenhouse gas emissions, indirect costs, and co-benefits) (Liel and Deierlein 2013; Hutt et al. 2016; Dong and Frangopol 2016; Haghpanah et al. 2017; ATC 2010a). On the other hand, when new design requirements are introduced to enhance life safety protection or secure emergency services, benefit analysis may be highly sensitive due to the incalculable value of human life and the immeasurable value of the services that save lives, and thus the focus shifts to the calculation of implementation costs and avoided casualty losses (Anagnos et al. 2016; Preston et al. 2019; Meade and Kulick 2007; DGS 2002). The goal of this review is to be comprehensive within the scope of our research questions, so there is no specific time period cutoff for publication.

Our review reveals that the key drivers of the cost-effectiveness of earthquake risk reduction are the building occupancy class (e.g., hospital, school, or residential and commercial), the location (e.g., high or moderate seismic hazard risk), and the performance target (e.g., life safety, immediate occupancy). In particular, decision makers often face a trade-off between the benefits and costs of a risk reduction measure, which increase with the performance target, and thus the highest level of performance is not always optimal in terms of benefits. Moreover, BCA results appear to be sensitive to other input assumptions, including the discount rate, planning horizon, and assumed cost of an earthquake risk reduction measure.

Our review culminates in a series of identified opportunities and challenges for research. We discuss the need for methods, data, and validation for building-level BCA, regional BCA, and the allocation of benefits and costs among building stakeholders. Moreover, we highlight the importance and underutilization of uncertainty quantification, including sensitivity analysis, uncertainty propagation, and stochastic methods. We also identify four understudied areas of high potential and impact: BCA for above-code design, BCA for code implementation, environmental benefits of seismic retrofits, and optimization of seismic retrofits with energy upgrades.

An important lesson from our review is that while BCA helps to enhance risk reduction decisions, final decisions should be made in a holistic context. The Unreinforced Policy Committee of Seattle (UPC 2017) stated that BCA provides valuable information for making policy recommendations. However, this analysis is not able to provide exact predictions of actual damage, nor provide exact estimates of benefits. Given these limitations, policy recommendations should be made based on all available information and within the context of the community rather than on a single analysis or model. Distributed BCA, which we identify as a research need, has the potential to support policy design by identifying potential equity issues arising from earthquake risk reduction. As with other available economic evaluation tools (Fung et al. 2022b), BCA has its strengths and weaknesses, and particular attention should be paid to the assumptions made to ensure the accuracy and reliability of such an analysis.

The next section describes the basic steps for performing a BCA. The section that follows presents our review of BCA for earthquake risk reduction, with a focus on analysis results, methods, and data sources. We then delineate the limitations of existing BCA approaches and research needs to improve the approaches for better accuracy and credibility. Our main contributions are presented in the following two sections, “New Methods and Research Needs” and “New Focus Areas and Research Needs,” which elaborate opportunities and challenges in the application of BCA for earthquake risk reduction. Finally, we conclude with a summary of lessons learned and practical recommendations for the implementation of BCA.

### Procedure for Benefit–Cost Analysis

#### Step 0: Set Analysis Parameters

Discount rate (r) is the rate of return used to discount future cash flows back to the present value. A typical discount rate is between 2% and 10% (Gibson et al. 2014). Planning horizon (T) is the future time period in years over which benefits and costs are counted. Planning horizon is typically between 50 and 75 years for new buildings (NIBS 2019) and 30 years for existing buildings after seismic retrofit (FEMA 2009).

The process of economic discounting for future damage (e.g., discount rate) tends to prioritize the well-being of individuals living today over those who will exist in the future (Lind 2007). From an equity perspective, all individuals should be treated as equals, regardless of whether they are currently alive or yet to be born (Lenton et al. 2023). Therefore, when the strategy being evaluated has long-term impacts on life and health, such as climate change mitigation, it is recommended to use nonconstant discount rates for analysis periods that extend beyond the planning horizon (Lind 2007), utilize distinct discount rates to adjust long-term benefits and investment costs (Welsh-Huggins and Liel 2018), or refrain from translating benefits into monetary terms (Lenton et al. 2023). Because earthquake risk reduction measures are effective within the relatively short planning horizon of buildings, applying the same discount rate to life-saving benefits and investment costs is preferable as indicated by many studies (Pate-Cornell 1984; Liel and Deierlein 2013; NIBS 2019).

#### Step 1: Estimate the Benefit, ${\text{B}}_{\text{i}}$, of Action i

This step requires first identifying assets that are sensitive to earthquakes and then estimating the relationship between the severity of expected losses and the level of ground shaking hazard. Benefits are estimated from the avoided losses under action i relative to the status quo

(1) $$B_{i}=\left[EAL_{0}-EAL_{i}\right]\sum_{t=1}^{T}{\left(1+r\right)}^{-t}$$

where t = time starting from the year that a mitigation action is taken; and $EAL_{j}$ = expected annual losses under action j, for j=0,….,I; where I is the set of actions, and is calculated as follows:

(2) $$EAL_{i}=\int_{0}^{\infty }l|dp(l)|$$

where p(l) = annual rate of exceedance for the loss l, given as follows (Krawinkler et al. 2006):

(3) $$p(l)=\int_{dm}\int_{edp}\int_{im}p(l|dm)dp(dm|edp)dp(edp|im)dp(im)$$

where p(x∣y) = exceedance probability of x given y (e.g., survival function, the complementary cumulative distribution function); dm = damage measure (e.g., damage state); edp = engineering demand parameters (e.g., maximum drift); im = intensity measure (e.g., peak ground acceleration); and p(im) = expected rate of return of the ground shaking hazard (e.g., hazard curve).

Direct benefits include avoided damage to buildings and contents, and avoided deaths and injuries. Indirect benefits may be economic or related to community resilience, social equity, and environmental sustainability, including avoided displacement and debris removal, loss of business or rental income, loss of life quality, loss of productivity, loss of customers, supply chain delays, reduction in employment, tax base, and affordable housing, among others (Fung et al. 2022b).

#### Step 2: Estimate the Cost, ${\text{C}}_{\text{i}}$, of Action i

The cost for alternative design is estimated as the difference in initial construction cost or life-cycle cost relative to the baseline. Initial construction cost may include material, labor, equipment costs, and contractor overhead and profits. Life-cycle cost is the total cost associated with building design and construction, building operation and maintenance, and building disposal at the end of the life cycle. The cost for seismic retrofit is a combination of structural and nonstructural improvement costs and may also include changes in maintenance costs (FEMA 2009; Fung et al. 2022b).

#### Step 3: Compare Benefits and Costs

Given estimates from Steps 1 and 2, one can distribute benefits and costs across stakeholders to obtain tiers of impacts (NIBS 2019; Fung et al. 2022a). Benefits and costs are compared using two metrics: benefit–cost ratio (BCR) and net present value (NPV)

(4) $${\text{BCR}}_{i}=B_{i}/C_{i}$$

(5) $${\text{NPV}}_{i}=B_{i}-C_{i}$$

where a ${\text{BCR}}_{i}>1>1$ or ${\text{NPV}}_{i}>0$ implies that the benefit of the action outweighs the cost.

Sensitivity analysis can be applied to examine whether the BCR shifts dramatically when inputs vary due to uncertainties in a building’s useful life, inflation rate, benefit and cost assumptions, hazard level, and model simulations. It is often helpful to determine the sensitivity range for each input and identify the inputs most important to BCR estimation (Gibson et al. 2014; Fung et al. 2022a).

### Use of Benefit–Cost Analysis in Earthquake Risk Reduction Studies

This section provides an overview of the methodologies employed in BCA studies and a summary of findings concerning the primary drivers of cost-effectiveness of earthquake risk reduction measures: code adoption, above-code design, and seismic retrofits. The literature selected here represents a collection of studies that share fundamental assumptions and research approaches and is not intended to be comprehensive. The conclusions about cost-effectiveness should be carefully interpreted because they depend on the assumptions made for benefit and cost estimation, the methods used to predict direct and indirect losses, and the reference cases. We especially encourage cautious interpretation of results from non-peer-reviewed studies.

#### Building Code Adoption

**Fig. 2.**
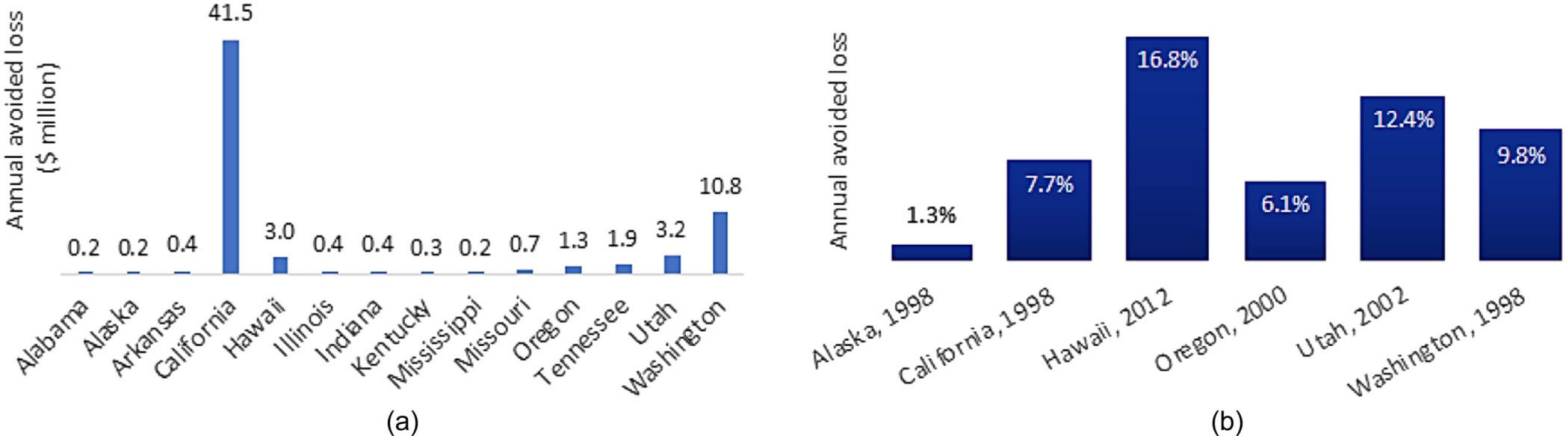
Benefit and cost for adopting new seismic codes relative to 1990s codes: (a) benefit of adopting 2000 I-Codes in 14 earthquake-prone states; and (b) impact of adoption year for 2000 I-Codes on annual avoided loss, relative to the baseline replacement value. (Data from FEMA 2020a, 2017.)

For new buildings, studies are regularly conducted to analyze the economic impacts of code changes. The economic impacts include reduced probabilities of property loss, death, and injury, population displacement, and business interruption in future earthquakes. Table 1 summarizes the literature on BCA for adopting new code requirements. At the national level, FEMA evaluated annual avoided losses for post-2000 buildings conforming to 2000 I-Codes (FEMA 2020a). The seismic requirements of 2000 I-Codes are equivalent to that of 1997 Uniform Building Code. The study combined damage functions from Hazus [a free geographic information system–based risk assessment tool developed by FEMA (2012)], parcel and building footprint data from multiple sources, and input from experts in building performance and building code history to develop detailed spatial loss estimates. The results show that annual avoided losses are significant for US states with high to moderate seismicity [Fig. 2(a)]. In highly seismic regions (Alaska, California, Hawaii, Oregon, Utah, and Washington states), an average 8% reduction in annual losses can be expected. The avoided losses are more pronounced in regions with higher seismic hazard (California), lower seismic design requirements (Hawaii), or both (Utah), as illustrated in Fig. 2(b). Similarly, the Multi-Hazard Mitigation Council (NIBS 2019) found that adopting the 2018 I-Codes for new construction in the 48 contiguous United States can result in a BCR of 12 compared to 1990s seismic codes. Specifically, implementing the 2018 I-Code requirements for earthquake can prevent annual property losses of $1,500 per building in 2018 US dollars, reduce annual deaths, injuries, and trauma-related losses by $800 per building, and lower annual business interruption losses by $2,000 per building (NIBS 2019). Other national-level studies focus on evaluating compliance costs. The goal of such studies is to demonstrate that the marginal cost of complying with the newer code is not very large relative to an earlier code (e.g., NAHB 2018).

Such studies naturally raise the question of the need to adopt new seismic standards in regions of moderate seismic risk. For instance, the middle Mississippi River Valley region experienced very large earthquakes in the past but no damaging earthquakes in recent decades (NEHRP 2013; Nordenson and Bell 2000). The National Earthquake Hazards Reduction Program (NEHRP 2013) assessed the benefits and costs of adopting the 2012 International Building Code (IBC) in Memphis, Tennessee, relative to the 1999 Standard Building Code (SBC). A major conclusion of their study is that the compliance costs are low, but the benefits associated with the improved design are significant. However, Stein et al. (2003) estimated that the total compliance cost for the 2000 IBC ($200 million/year in 2001 US dollars) is an order of magnitude greater than the total benefit ($17 million/year) and argued that buildings in Memphis should not be designed to the same level as in California because of lower seismic hazard. Ryu et al. (2010) also showed that designing new commercial buildings in Memphis to the 2003 IBC, 2006 IBC, or 2009 NEHRP provisions has little effect on expected annual losses (EALs) relative to the 1999 SBC. The controversy between NEHRP (2013) and the two studies is due to different versions of seismic hazard maps used, building types analyzed, and benefit elements considered. Similar debates exist in Charleston, South Carolina, and Boston, where recent seismic activity is minor but magnitude 7 or larger earthquakes struck the regions in the past (Nordenson and Bell 2000; Nikellis et al. 2019; Joyner and Sasani 2018).

A potential gap in such studies is the absence of co-benefits, which accrue even in the absence of a hazard event during the planning period (Fung and Helgeson 2017). A few studies have evaluated the co-benefit of seismic codes on wind mitigation. Nikellis et al. (2019) analyzed steel moment frame (SMF) office buildings in two US cities and found that ignoring wind-induced losses in Los Angeles can lead to a 32%–62% underestimation of EAL for 40-story buildings. Ignoring earthquake-induced losses in Charleston, South Carolina, can result in a 33% and 29% under-estimation of EAL for 30-story and 40-story buildings, respectively. However, Joyner and Sasani (2018) indicated that the co-benefit is negligible in earthquake or wind-controlled regions. Wind damage accounts for 5% of the total EAL for a 7-story concrete building in San Francisco (earthquake-controlled). Earthquake damage accounts for 1% of the total EAL for a 7-story concrete building in Boston (wind-controlled). The controversy between the two studies is mainly due to different building heights and structural types analyzed, and further research is needed.

Unlike many studies that assess benefits based on predicted building performance, a few studies have used historical insurance data to evaluate avoided losses due to the implementation of a building code (e.g., Simmons et al. 2020, 2018). This approach compares paid insured losses before and after code implementation, facilitating regional-level impact assessment. A caveat is that buildings built after the enactment of the code are assumed to comply with the code, whereas in practice, buildings may be built to lower or higher standards, depending on code enforcement, quality control, and owner requirements for safety and resilience. Moreover, this method is more suitable for frequent natural hazard events such as hurricanes because it requires comparable events in intensity or magnitude before and after code implementation.

#### Above-Code Seismic Design

Several studies have assessed the benefits and costs of above-code seismic design (Table 1) and found it to be a cost-effective option (NIBS 2019; Kutanis and Boru 2014). Specifically, the MultiHazard Mitigation Council (NIBS 2019) estimated that buildings above the 2015 IBC requirements could result in a national average BCR of 4, relative to 1990s seismic codes, meaning that $4 can be saved for every $1 spent to make new buildings stronger and stiffer. To achieve greater building strength than required by the 2015 IBC, the Multi-Hazard Mitigation Council assigned the buildings a higher importance factor than specified by the 2015 IBC. The vulnerability functions used in Hazus were also modified to reflect the increased strength and stiffness of the buildings. Likewise, Kutanis and Boru (2014) recommended adjusting the performance target of residential buildings to immediate occupancy in Turkey, where 71% of the land is located in high seismicity zones. Kutanis and Boru (2014) designed six benchmark residential buildings of three heights and two structural systems (infilled frame and dual system), and estimated that construction costs could increase by 4.2%–11.2% for 3-story buildings, 21.2%–28.8% for 6-story buildings, and 20.7%–27.4% for 10-story buildings built to the immediate occupancy level relative to the life safety level. The expected annual cost increase for new construction is comparable to the historical annual loss from earthquakes, meaning that the BCR is greater than 1, assuming no loss in the immediate occupancy scenario.

#### Seismic Retrofits for Older Buildings

There is an extensive literature on evaluating the economic value of seismic retrofits for existing buildings. A major focus is on bringing existing residential and commercial buildings up to the life safety standard. Another focus is on improving the performance of hospitals and schools to the immediate occupancy level in the event of a major earthquake. Given that much of the variation is across structural systems and risk categories, we present our review by building type. Table 2 summarizes the methods employed in the literature for evaluating retrofit strategies applicable to hospitals, schools, and residential and commercial buildings.

##### Hospitals (Risk Category IV)

Following the 1994 Northridge earthquake, California passed Senate Bill (SB) 1953, which required that the state’s hospitals not only maintain structural integrity but also continue operations after an earthquake. Meade and Kulick (2007) estimated that the cost for 2,484 hospitals to comply with SB 1953 could be as high as $41.7 billion in 2006 US dollars. Preston et al. (2019) updated the cost analysis with respect to the 2030 deadline for ensuring post-earthquake operational performance. The estimated compliance costs are still outstanding between $34 billion and $143 billion in 2019 US dollars. These figures demonstrate that improving the performance of existing hospitals to the immediate occupancy level can incur significant costs. However, investing in higher performance can shorten payback periods and increase the overall benefit of risk mitigation. Ghesquiere et al. (2006) estimated that for hospitals in Bogota DC, Colombia, the annual rate of return could be 19.1% for basic structural reinforcement, but 32.8% for comprehensive mitigation that enables hospitals to remain functional during and immediately after a seismic event. Notably, the avoided deaths due to retrofits include not only patients and staff at the hospital but also lives saved because hospitals are able to provide emergency service to affected populations after earthquakes (Ghesquiere et al. 2006).

##### Schools (Risk Category III)

The California Department of General Services was mandated in 1999 to inventory statewide concrete tilt-up and non-wood-frame school buildings that fail to meet the minimum requirements of the 1976 Uniform Building Code (UBC) and to improve the seismic safety of these vulnerable buildings (DGS 2002). The department estimated that it could cost $4.09 billion and $4.57 billion in 2002 US dollars, respectively, to bring 7,537 school buildings up to life safety and damage control performance levels, taking into account engineering evaluation costs, program administration costs (20% of the total costs), and structural and nonstructural retrofit costs. Structural retrofit costs were estimated using the FEMA 156 methodology, which provides a cost inventory for 2,100 projects with different structural types and performance targets across the United States (FEMA 1994; Fung et al. 2020). Nonstructural costs were estimated based on the assumed cost per square foot, which does not vary by building type and performance level. Although this study did not provide detailed estimates for each building, it suggests that retrofitting buildings to a higher level of performance than life safety may not result in significant cost increases.

The cost-effectiveness of upgrading school buildings to a higher performance level has been evaluated in several studies. Haghpanah et al. (2017) found that a 4-story reinforced concrete school building in California could be upgraded to an immediate occupancy level of performance by installing base isolation. Although base isolation has a high initial cost, it can significantly reduce economic and casualty losses in future earthquakes, making it cost-effective in the long run. Likewise, Carofilis et al. (2020) analyzed three types of school buildings designed and constructed in Italy before the 1970s. The objective of the retrofit is to bring the schools up to the current Italian building code. The analysis results indicate that it is cost-effective to strengthen unreinforced masonry (URM) buildings by attaching carbon fiber–reinforced polymer (CFRP) strips to both sides of masonry piers and spandrels. The payback period is 32–39 years at a discount rate of 0%–1%. In addition, it could be cost-efficient to strengthen precast concrete buildings by using steel dowels and viscous dampers together. The payback period is 56–83 years. However, other strategies are not economically feasible due to limited performance improvement or high retrofit costs, as summarized in Table 3.

##### Steel Buildings

SMFs are widely used as the seismic force-resisting system for tall buildings in California (Hamburger and Malley 2019). However, the 1994 Northridge earthquake revealed a deficiency in the welded moment connections and fostered the development of SMF evaluation and strengthening methods (Hamburger and Malley 2019; Bonowitz and Maison 2003). Hutt et al. (2019) indicated that the collapse probability of older SMF buildings is 28 times greater than that of modern code-conforming SMF buildings.

The literature on seismic retrofitting of SMF buildings is scarce, with a few studies assessing the benefits of retrofitting. Hutt et al. (2016) analyzed a 40-story SMF building designed to the 1973 UBC in San Francisco. Three strategies were devised to improve the structural and nonstructural systems of the building: (1) adding an elastic spine frame with steel bracing, (2) installing base isolation systems at ground level, and (3) using earthquake-resilient nonstructural components. Using the PACT software, Hutt et al. (2016) estimated the direct economic loss due to structural and nonstructural damage. The repair time was estimated in accordance with the Resilience-based Earthquake Design Initiative (REDi) guidelines (Almufti and Willford 2013). The three retrofit strategies can reduce the economic loss to 25%, 7%, and 23% of the building’s replacement value (34% in the status quo), and reduce repair time to 72, 59, and 32 weeks, respectively (87 weeks in the status quo). Using these strategies together can further improve building performance. In particular, Strategies 2 and 3 together can reduce the loss to a minimum of 3% and the repair time to less than 1 day. Similarly, Dong and Frangopol (2016) found that installing base isolation can reduce 65% of repair cost, 75% of fatality loss, 36%–55% of downtime, and 43% of carbon emissions for a 3-story SMF building in Los Angeles under the simulated 1940 El Centro earthquake ground motion. Overall, these studies show significant benefits from seismic retrofitting, but future research should compare the benefits with retrofit costs to provide a more complete picture.

##### Reinforced Concrete Buildings

Nonductile concrete buildings were largely constructed in California prior to 1972. The design and construction methods were significantly improved in the 1976 UBC, motivated by damage to this structural type in the 1971 San Fernando earthquake (ATC 2010b). The literature indicated that the collapse probability of nonductile concrete buildings is 10–80 times greater than that of modern code-conforming concrete buildings (Liel and Deierlein 2013; Liel et al. 2011). Therefore, many studies sought to develop cost-effective retrofit strategies for older concrete buildings.

Liel and Deierlein (2013) evaluated four retrofit strategies for nonductile concrete buildings in Los Angeles: (1) replacing with modern buildings designed to 2005 code provisions, (2) jacketing existing columns with reinforced concrete, (3) adding wall piers, and (4) wrapping existing columns with fiber-reinforced polymer (FRP). The authors designed eight archetype concrete office buildings based on the 1976 UBC, including four building heights (2, 4, 8, and 12 stories) and two structural systems (space frame and perimeter frame). Replacement costs were estimated using the *RSMeans Estimating Handbook*, and retrofit costs were estimated using the FEMA 156 document. The benefits account for reduced repair costs and casualties over 50 years. The results show that Strategy 1 is cost-effective for the 2-story space frame. Strategies 2 and 3 are cost-efficient for 4- and 8-story space frames. Overall, the cost-effectiveness of retrofit strategies depends on building height, structural system, and the cause of the structural deficiency.

Kappos and Dimitrakopoulos (2008) investigated the optimal level of seismic retrofits for older concrete buildings in Thessaloniki, Greece. The optimal level is interpreted as the retrofit state that yields the minimum life-cycle cost. The life-cycle cost is a sum of initial retrofit costs and EAL over 40 years. The highest retrofit level is full retrofit, denoted by 1, which upgrades buildings designed under the 1959 Greek code to modern seismic codes for new construction. The lowest retrofit level is no action, denoted by 0. The results show that the optimal retrofit levels for 2-story infilled-frame buildings in Seismic Hazard Regions VI–VIII are 0.4, 0.5, and 0.7, respectively, when casualties are ignored, and 0.5, 0.8, and 0.8, respectively, when casualties are considered. The optimal levels for 4-story buildings are nearly zero, suggesting that seismic retrofits are not cost-effective for midrise buildings. The optimal levels for 9-story buildings are between those of 2- and 4-story buildings. Likewise, Vitiello et al. (2017) analyzed five retrofit methods for a 5-story concrete building in L’Aquila, Italy, and found that the optimal retrofit level depends on retrofit method, building height, location (seismic hazard), and structural system.

##### Wood-Frame Buildings

Conventional wood light frames were largely used in single-family houses and multifamily housing in the United States. In California, wood-frame apartments built between 1920 and 1970 often contain large openings at the ground level due to garages or commercial space, causing weak or soft-story problems, which are a prevalent failure mechanism observed in the 1989 Loma Prieta and 1994 Northridge earthquakes (ATC 2010a). Several California cities have initiated a mandatory retrofit program for soft-story buildings to reduce collapse risks and prevent casualties in future earthquakes (Zhang et al. 2022).

To inform the development of the retrofit program, the Applied Technology Council evaluated three retrofit schemes for four representative residential buildings in San Francisco. Benefits were measured as avoided structural and contents losses during a magnitude 7.2 San Andreas Fault earthquake (ATC 2010a). Scheme 1 targets the life safety performance objective. Steel moment frames and limited shear walls are installed in the soft story. Scheme 2 adds steel moment frames and larger shear walls to the soft story to prevent demolition of the building after an earthquake and to provide protection in addition to that of Scheme 1. Scheme 3 targets the immediate occupancy performance objective. Steel cantilevered columns and larger shear walls are installed to strengthen the soft story. The results show that the avoided losses exceed the retrofit costs of the three schemes in a magnitude 7.2 earthquake. However, it is important to note that the protection provided by retrofitting is limited. In an extremely large earthquake, buildings with and without retrofitting can be expected to suffer similar levels of severe damage and repair costs. Therefore, the economic benefit of retrofitting may decrease as the magnitude of an earthquake increases.

Similarly, Porter et al. (2006) conducted a BCA for retrofitting wood-frame residential buildings to comply with the 1997 UBC in California. To assess the benefits of retrofit schemes, the authors determined the probabilistic site hazard at the zip code level and developed assembly-based vulnerability (ABV) models to predict building damage and repair costs. Two retrofit schemes were evaluated for a 3-story apartment building constructed in the 1960s. Scheme 1 installs steel moment frames at garage openings. Scheme 2 adds structural sheathing to the center longitudinal wall on the ground floor. The results show that both schemes are cost-effective in zip codes near earthquake faults and on soft soils. However, the estimated benefits only account for building damage and repair costs, which can be greater when other potential losses are considered, such as loss of contents, loss of function, and human injury (Porter et al. 2006).

##### Unreinforced Masonry Buildings

Unreinforced masonry buildings were largely constructed in California before the 1933 Long Beach earthquake. The buildings perform poorly in earthquakes due to the brittle nature of the material along with inadequate steel reinforcement or insufficient structural connections between the building’s walls, parapets, and roof. Construction of URM buildings was prohibited after the Field Act. In 1986, California mandated all jurisdictions in Seismic Zone 4 to develop a risk reduction program for existing URM buildings (SSC 1992). However, in other regions, such as Seattle, Washington, and Portland, Oregon, implementing a mandatory retrofit program for URM buildings has proven challenging, primarily due to high retrofit costs (UPC 2017; PBEM 2017).

Gibson et al. (2014) analyzed two retrofit options for URM buildings in Seattle: parapet bracing and the bolts-plus method. The bolts-plus method was adopted by the City of San Francisco in 1992 as part of Ordinance 225–92. It involves the installation of shear and tension anchors in the roof and floors, and the bracing of the unreinforced masonry bearing walls when required. The results show that replacement and demolition are not as economical as parapet bracing unless the existing URM building is in worse than average condition. In addition, the benefit (reduced EAL) of the bolts-plus method is greater than parapet bracing but is marginal relative to its implementation cost (8%). This is mainly because the probability of building collapse in the city is very low even without retrofitting.

Similarly, Paxton et al. (2017) evaluated three retrofit strategies for URM buildings in downtown Victoria, British Columbia, Canada: parapet bracing, partial retrofit, and full retrofit. Partial retrofit adds tension anchorages to all floor-to-wall interfaces and extremely slender walls, and strengthens out-of-plane bracing in addition to bracing parapet walls. Full retrofit attaches shear anchorages to all diaphragms, increases the size of vertical supports, and strengthens in-plane walls and diaphragms in addition to the reinforcement method used for partial retrofit. The results show that parapet bracing and partial retrofit are cost-effective in enhancing the seismic performance of URM buildings. Full retrofit is not economically feasible, but it results in the lowest economic losses and least casualties in the aftermath of earthquakes. Overall, these studies indicate that it may not be economically feasible to adopt the highest level of retrofit for URM buildings in regions of moderate seismic risk.

Tables 3 and 4 summarize the BCA results for seismic retrofit methods found in the literature. In particular, Table 3 focuses on retrofitting existing buildings to exceed life safety performance requirements. This can provide valuable, though not exhaustive, insights on how to achieve post-disaster functional recovery objectives in a cost-effective manner. Table 4 delves into retrofit strategies that bring existing buildings to more current performance requirements for life safety. This can potentially inform ongoing risk reduction efforts for the most seismically vulnerable buildings.

### New Methods and Research Needs

Based on the literature review in the preceding, we highlight opportunities for research into new methods for the use of BCA in earthquake risk reduction. We present opportunities and challenges across two broad analysis categories (BCA for representative buildings and BCA for buildings at the regional scale) and post-analysis best practices (distributed BCA and uncertainty quantification).

#### Benefit–Cost Analysis for Representative Buildings

Building-level analysis often requires information on site conditions, structural and nonstructural components, contents value, and number of occupants. Because detailed hazard, building, and occupancy information is supplied to the analysis, detailed estimates can be obtained for building damage, collapse probability, and casualties. Methods for quantifying these direct impacts are well developed and validated by many studies. In contrast, indirect economic impacts are less readily assessed in the literature due to lack of data or difficulty in quantifying the losses as well as gaps in estimating recovery time (Fung et al. 2022b). This can result in underestimated economic benefits of risk reduction measures, and thus discourage building owners from adopting such measures (Goettel 2016; Gibson et al. 2014).

Building-level analysis often begins with the development of vulnerability models that consider various levels of earthquake intensity. The models are developed based on empirical data, experimental tests, statistical analysis, or expert judgment. However, expert opinion and methods that are difficult to verify should be used with caution (Porter et al. 2006). Validating models with post-disaster building damage records can improve the accuracy of damage predictions (Cook et al. 2021; Baker et al. 2016). Earlier studies determined damage state probabilities based on building response characteristics (e.g., peak acceleration, peak drift ratio), while more recent studies have used the Performance-Based Earthquake Engineering (PBEE) approach to analyze damage states of single components and aggregate component-level results to the whole building. The PBEE approach enhances the analyst’s ability to predict damage state probabilities and probabilistic repair time and cost for individual buildings.

First, the data required to quantify various indirect benefits are scarce, which hinders the practice of incorporating indirect benefits into BCA (Goettel 2016; Gibson et al. 2014; Fung et al. 2022b). However, reducing indirect losses such as business interruption and displacement is part of the objective of resilient design. When possible, the co-benefits of risk reduction strategies should be accounted for; that is, the benefits accrued when there is no damage event during the analysis period. These include, but are not limited to, extending the life of existing structures (Keskin et al. 2021), improving environmental sustainability (Wei et al. 2016), reducing insurance premiums (Marshall 2018), increasing the market value of buildings, improving historic preservation, and maintaining the visual character of communities (Gibson et al. 2014). Second, there is a need to further improve the methods used to estimate recovery time. Recovery time is a crucial parameter for economic evaluation because it relates to business interruption, replacement and relocation, and critical services based on the building’s use category. The FEMA P-58 database is the most comprehensive resource currently available, providing extensive data on component damage and consequences to aid in recovery time estimation. However, the database does not cover all components used in US construction practice (Cook at al. 2022). Additionally, there is limited understanding of how external lifeline infrastructures may influence individual buildings, and this factor represents a significant source of uncertainty in estimating the time for buildings that rely on these external facilities to recover their services (Cook et al. 2022).

#### Benefit–Cost Analysis for Buildings at the Regional Level

Regional-level analysis is crucial for policymakers to understand the large-scale social and economic impacts of the implemented standards and policies. In these analyses, benefit and cost outcomes are often aggregated for various building groups. Building groups are classified by building age, height, structural system, occupancy class, risk category, design level, performance objective, and/or seismic zone. Studies based in the United States have mostly relied on Hazus to estimate repair or replacement costs for structures and nonstructural components, contents losses, relocation costs, business and rental income losses. When using Hazus, it is important to supply detailed building information (FEMA 2020a), validated vulnerability models (Gibson et al. 2014), and the most recent hazard maps to the software (Ryu et al. 2010).

More recently, researchers have developed web-based software platforms to support advanced regional hazard and vulnerability analysis. The Interdependent Networked Community Resilience Modeling Environment (IN-CORE), created by the Center of Excellence for Risk-Based Community Resilience Planning (CoE), allows users to apply various hazards to infrastructure in selected areas, propagating the effect of physical infrastructure damage and loss of functionality to social and economic impacts (NIST 2020). The Natural Hazards Engineering Research Infrastructure’s Computational Modeling and Simulation Center (NHERI SimCenter) is creating a suite of computational workflows to simulate earthquake and hurricane effects on communities (Deierlein and Zsarnóczay 2021; Deierlein et al. 2020).

These platforms offer three key advantages compared with Hazus: (1) leveraging cloud computing techniques and high-performance computational resources to handle large data sets, (2) facilitating high-resolution simulation by combining the detailed assessment of individual facilities and comprehensive regional-scale simulation of natural hazard effects, and (3) using workflows to link numerous software applications, libraries, and databases, and allowing the integration of user-supplied workflow components, such as user-compiled building spatial data (Deierlein and Zsarnóczay 2021; Deierlein et al. 2020).

Using the NHERI SimCenter platform, Hulsey et al. (2022) studied the impact of safety cordons on the recovery of office space in downtown San Francisco. Xiong et al. (2019) also used the SimCenter platform to model the recovery process of nearly 70,000 residential buildings in Beijing, accounting for the impeding factor of labor constraints. Wang et al. (2021) extracted building information from street and satellite images using the SimCenter platform, and incorporated the data into the workflow to assess city-scale seismic risks.

The primary challenge in using Hazus to assess regional seismic risk is to modify or replace default vulnerability functions so as to correctly represent modern code-conforming buildings. The default seismic vulnerability functions in Hazus are based on the performance of the general US building stock through the 1990s (FEMA 2012). Modern code requirements and construction practices are not integrated into the risk analysis framework. However, it is expensive to create new vulnerability functions that reflect modern design practices for all US building types, which have nearly 700,000 categories (NIBS 2019). Therefore, some studies have used approximation methods that modify default vulnerability functions to characterize modern buildings or above-code designs, which is not a perfect solution but is efficient (e.g., NIBS 2019). In addition, while Hazus offers great flexibility for users to modify model parameters based on research needs, some fixed parameters can have a large impact on loss estimation, such as modifiers for occupancy class, damage state, and building downtime. These modifiers are determined by engineering judgment without rigorous validation, which may introduce large uncertainties into loss estimation.

Advanced regional simulation approaches such as the NHERI SimCenter and IN-CORE can overcome the constraints of Hazus, but the lack of an inventory of building characteristics presents a new challenge to the quantification of regional seismic risk (Deierlein et al. 2020). Moreover, recent improvements in these approaches are limited to building performance evaluation and loss estimation, while construction cost increases, which are important for BCA and decision-making, are assumed to be insignificant and rarely investigated. Another research opportunity is to develop computational methods for estimating costs and losses across a wide range of direct and indirect impact categories that are interoperable across systems, such as the NHERI SimCenter and IN-CORE.

#### Distributed BCA: Allocation of Benefits and Costs to Stakeholders

In principle, BCA should take a holistic perspective that reflects the impact of a risk reduction decision on society as a whole. In practice, there are often implicit boundaries placed on the problem for tractability and measurement of losses. An alternative approach is to explicitly model the BCA perspective to properly bound the problem. An example is the owner-focused BCA method proposed by Cutfield and Ma (2015). This method entails distinguishing and identifying the cash flows between the building owner and external entities, with a specific focus on those cash flows that may vary depending on whether the building undergoes improvements. More broadly, a holistic BCA can distribute benefits and costs across relevant stakeholders, either by separating BCAs for each stakeholder (Fung et al. 2022a) or by using a benefit-transfer matrix on the community-level BCA (NIBS 2019). This step may be crucial for making a business case because it helps identify winners and losers from a risk reduction decision and thus can assist communities in prioritizing resources such as outreach and financial support.

To our knowledge, the best and only source of data for holistic, distributed BCA is the benefit-transfer matrix developed by the Multi-Hazard Mitigation Council (NIBS 2019), shown in Table 5. The benefit-transfer matrix can be used to allocate estimated costs and benefits to five closely involved stakeholder groups: developers, building owners (title holders), lenders, tenants, and communities (e.g., visitors, emergency service providers), as illustrated in Fung et al. (2022a, b). An important caveat, however, is that the benefit-transfer matrix was developed through an expert elicitation process and the underlying assumptions (summarized in Table 5) are not fully transparent or validated. While it is a valuable source of data, the lack of transparency and validation may limit its application in practice. We therefore note future research opportunities to validate the benefit-transfer matrix, provide a range of values to characterize uncertainties, or add other relevant stakeholders to this matrix.

#### Uncertainty Quantification for the Benefit-to-Cost Ratio

It is a recommended practice to quantify uncertainties associated with loss prediction and cost estimation. Several methods are available:

- Sensitivity analysis measures the influence of inputs on BCR. The examined inputs include discount rate, analysis period, hazard level, investment costs, building and content values, casualty costs, and business interruption costs if available (Gibson et al. 2014; Goettel 2016). While it is broadly acknowledged that BCR results are sensitive to input assumptions, sensitivity analysis is not consistently incorporated in the literature.
- Uncertainty propagation methods estimate the probability distribution of BCR. The uncertainties in ground motion, structural model, collapse capacity, repair method and cost, and construction cost are often considered in the literature. Uncertainty propagation methods include Monte Carlo simulation, Latin hypercube simulation, moment matching, and first-order second-moment analysis. While such methods are well established, uncertainty propagation is rarely incorporated in BCA studies due to the complexity of both modeling and aggregating individual uncertainties and the potential to amplify noise relative to signal due to such complexities.
- The random variable method uses the outputs of the earthquake risk model to obtain a random variable for (structural) losses. This yields a random variable for benefits (as the difference in random losses with and without mitigation), which can be used to derive an exceedance curve for BCR (Ghesquiere et al. 2006; Cardona et al. 2008). This method is underexplored and underused.
- The variance method computes the variance of the difference in EAL between the enhanced building and the status quo based on the raw first and second moments of each EAL. The variance of avoided losses can be used as a measure of variation of economic losses that depend on avoided losses (Fung et al. 2022b). This method is simpler than the random variable method, though to our knowledge has not been used in the literature.

### New Focus Areas and Research Needs

Finally, we identify four topics that require further research: BCA for above-code design (particularly functional recovery); BCA for code implementation (specifically code enforcement); environmental benefits of seismic retrofits; and benefits of combined seismic and energy retrofits.

#### Benefits and Costs of Above-Code Seismic Design

Building codes provide minimum seismic design requirements that focus primarily on saving lives and reducing injuries, rather than ensuring that buildings are functional, habitable, or repairable after a seismic event (NIST 2021). Designing buildings beyond codes with higher performance objectives can prevent lengthy and costly repairs and decrease the likelihood of economic and social disruption. However, much of the literature to date has focused on bringing new construction up to code. The economic implications of above-code seismic design are not well documented and quantified.

Functional recovery is a new design paradigm that aims to prevent lengthy repairs of buildings after a natural hazard event (NIST 2021). Realizing functional recovery for individual buildings and infrastructure systems is a mechanism for achieving community-wide resilience (EERI 2019). Although some studies have evaluated the impact of above-code design on downtime using Hazus, the evaluations are based on regional average building characteristics, and building-specific attributes that are crucial for individual buildings to attain desired recovery performance are not considered (e.g., NIBS 2019). As new approaches are developed to estimate recovery time for single buildings (e.g., FEMA P-58, REDi, ATC-138), designing buildings to achieve desired functional recovery performance targets has become possible (Cook et al. 2022; Hutt et al. 2022; Terzic and Villanueva 2021).

In addition, Fung et al. (2022b) developed a BCA framework to support the economic evaluation of recovery-based design. The framework considers direct economic impacts and indirect economic impacts due to loss of building functions, such as population displacement, business interruption, supply chain disruption, and loss of life quality, while highlighting gaps in methods and data availability. Previous studies have suggested that indirect impacts are equally important as direct impacts from seismic events (Toyoda 2008; Petak and Elahi 2001). Table 6 presents an example application of the framework for three functional recovery objectives.

First, design standards for functional recovery are currently under development (NIST 2021). The benefits and costs of recovery-based design can vary significantly depending on the strategy employed to achieve the recovery time goal. However, this offers an opportunity for using BCA to explore the driving factors for the cost-effectiveness of resilient design. Second, functional recovery can affect stakeholders with conflicting goals if tenants rather than owners benefit from recovery of function through reductions in business interruption and displacement costs (Fung et al. 2022b).

It is important to understand the mechanism by which benefits and costs are distributed among stakeholders and to utilize this mechanism to design buildings that are cost-effective to various stakeholders. Third, there is no public database that provides standard cost premiums for seismically rated nonstructural equipment, anchorage, bracing, and isolators (Tokas 2011), making cost estimates highly sensitive to market conditions and contractor profit.

#### Benefits and Costs of Building Code Enforcement

Regardless of the building design criteria, there are practical challenges to implementing building codes. Adopting codes on a regular cycle incurs costs for staffing and public meetings, as well as the purchase of new code books for building departments and training for code officials (FEMA 2020a; NEEP 2021). Implementing new codes also requires extensive education and training for contractors, installers, and inspectors to ensure that code requirements are duly and properly followed (FEMA 2020a; NEEP 2021). Moreover, incentivizing compliance by offering design and construction grants, cost reimbursements, tax benefits, or fee waivers for plan review, building permits, and inspections can further increase the cost of code implementation (MultiHazard Mitigation Council 2020). Most of the costs are borne by local governments, except for plan review and site inspection, which are fully or partially funded by building owners through permit fees (FEMA 2020a, 1998).

Even though many states have adopted a modern building code, the effectiveness of code implementation varies widely among local jurisdictions, affecting the level of protection that building codes are able to provide in newly constructed assets (FEMA 2020a). Past natural disasters have demonstrated that poor code enforcement can jeopardize building integrity and occupant safety (ISO 2019; FEMA 1998). Porter et al. (2006) estimated that compared to typical and superior constructions, poor construction can increase the median lifetime repair cost of wood-frame small houses in California by $3,000 and $5,400, respectively, in 2002 US dollars, wood-frame townhouses by $1,400 and $1,700, and wood-frame apartments by $8,700 and $13,000, considering a useful life of 30 years and earthquake hazard only. Poor, typical, and superior constructions were classified by the strength of the structural member relative to the strength in laboratory conditions. Poor constructions have a strength between 60% and 85%; typical constructions have a strength between 85% and 100%; and superior constructions have a strength comparable to laboratory test results for high-quality specimens (Porter et al. 2006).

The costs and benefits of code enforcement are rarely assessed in BCA studies due to a lack of data and methods to quantify the monetary values. The insurance industry is using the Building Code Effectiveness Grading Schedule (BCEGS) to assess the effectiveness of communities in enforcing code requirements for earthquake and wind design (ISO 2019, 2018). The BCEGS program assigns a grade of 1 (best enforcement) to 10 (no enforcement) to municipalities every 5 years based on 27 indicators, including enforced building code edition, training and certification of code enforcers, contractor and builder licensing and bonding, number of inspection permits issued, and level of detail of plan review and inspection (ISO 2019, 2018). Policyholders may receive a premium discount in communities that actively enforce building codes, and the amount of discount could be a good predictor for code enforcement benefits. The BCEGS rating only applies to residential and commercial buildings in municipalities that participate in this program. In addition, the BCEGS database provides expenditure data collected from state and local building code enforcement departments (ISO 2019), which could be a good source for researchers to estimate code enforcement costs. However, this is an understudied area that requires further research.

#### Environmental Benefits of Seismic Retrofits

The environmental benefits of seismic retrofits are rarely assessed or included in BCA. However, this does not mean that the benefits are negligible. Seismic retrofits can significantly reduce environmental impacts by reducing the likelihood of repairing, reconstructing, or demolishing a building after an earthquake (FEMA 2018; Ribakov et al. 2018; Comber et al. 2012). Comber et al. (2012) estimated that structural retrofitting of a 1960s research laboratory in California can reduce the carbon footprint by 48% (3,514 tons of carbon dioxide equivalent, or t CO_2_e), and structural and nonstructural retrofits collectively can reduce the carbon footprint by 77% (5,636 t CO_2_e). In highly seismic regions, seismic retrofits can prevent the same order of carbon emissions as energy upgrades (Comber et al. 2012).

On the other hand, building repair and demolition can have large environmental impacts (Gonzalez et al. 2022; Keskin et al. 2021). Following the 2010–2011 Canterbury earthquake, 7,500 dwellings and 1,400 commercial properties were demolished in Christchurch, New Zealand. Gonzalez et al. (2022) estimated that the demolition of 142 of the reinforced concrete buildings can lead to 159,966 t CO_2_e of carbon emissions. Demolishing newly constructed buildings can result in a greater environmental burden. Of the 142 buildings, 40% of the carbon emissions come from 30% of the buildings that were demolished less than halfway through their service life. This suggests that earlier retrofit intervention is beneficial for reducing environmental impacts.

There are three main challenges associated with quantifying the environmental benefits of seismic retrofits. First, currently available tools have a large discrepancy in environmental impact estimation due to different data collection methods and different inventories of carbon and energy for building materials, construction, transportation, maintenance, demolition, disposal, and recycling (Martinez-Rocamora et al. 2016). This implies large uncertainties in the quantification of environmental impacts and poses a challenge for incorporating environmental benefits into BCA. Second, the environmental impacts of seismic retrofits themselves are not negligible (Menna et al. 2022; Napolano et al. 2015). However, few studies investigated the trade-off between the environmental impacts of seismic retrofits relative to the avoided losses (Wei et al. 2016). The methodology for evaluating the trade-off for new building design has been discussed in the literature (e.g., Welsh-Huggins and Liel 2018), which could provide useful insights for existing buildings. Third, retrofits may extend the useful physical or economic life of buildings due to upgrades, alterations, or replacements to structural frames and nonstructural systems (Bonowitz et al. 2014). However, many studies ignore such effects and assume a useful life of 30 to 100 years based on statistical averages or initial building design (Table 2). Only a small number of studies consulted managers who are responsible for the retrofit project about the expected service life of the building (e.g., Keskin et al. 2021). In summary, with increasing attention on climate adaptation worldwide, there is a need to address these open questions on incorporating environmental benefits of seismic retrofits into BCA.

#### Optimization of Combined Seismic and Energy Retrofits

Recent studies also explored the use of combined seismic and energy retrofits for existing buildings. It is not surprising that the two forms of retrofits are often performed simultaneously for large commercial buildings in earthquake-prone regions because this can minimize displacement and business interruption compared to performing retrofits separately (Griffin 2017). In addition, combined retrofits can reduce payback periods and help cities meet decarbonization goals. Pohoryles et al. (2020) estimated that combined retrofits can reduce payback periods by up to 10 years compared to energy retrofits alone in moderate to high seismicity regions. Even in low seismicity regions, payback periods can be significantly reduced for the oldest buildings. Moreover, applying combined retrofits to 3% of the existing building stock in 20 European cities can reduce carbon emissions by 26.8%–37.7% in a decade, aligned with the European decarbonization goal of a 30% reduction by 2030 (Pohoryles et al. 2020). Mauro et al. (2017) also highlighted the importance of integrated design for seismic and energy retrofits because optimizing energy retrofits alone can diminish overall cost-efficiency.

Two methods have been proposed to optimize combined seismic and energy retrofits. One method focuses on minimizing life-cycle costs, which sums retrofit costs, seismic losses, and energy costs throughout a building’s service life. Caution should be exercised when seeking the lowest life-cycle cost because the lowest cost may correspond to the worst building performance. Gencturk et al. (2016) noted that high performance is often associated with high retrofit costs, which ultimately lead to high life-cycle costs. Another method is to maximize the benefit up to the point in which an additional investment does not result in increased performance. The building performance is measured by the green and resilient indicator (GRI), which is a ratio of expected annual seismic losses and energy costs to building replacement value (Calvi et al. 2016). A lower GRI value indicates better building performance.

There are two major challenges associated with retrofit optimization. First, energy consumption data (i.e., as energy use per square foot per year) for pre- and post-energy upgrades are rarely collected in the United States; instead, the outcomes of energy retrofits are always estimated through computer modeling (Griffin 2017). However, the outcomes of multiperformance retrofits cannot be fully modeled with existing tools designed for conventional energy retrofits (Griffin 2017). This may affect energy cost estimates and ultimately optimization results for combined retrofits. Second, retrofit costs are self-reported by building owners in the United States, which may or may not include retrofit grants, tax credits, and other financial incentives received. This causes large uncertainties in comparing data across buildings (Griffin 2017). While combined retrofits can increase cost-effectiveness, redesign and reconstruction could be more effective for buildings in very poor condition (Ferreira et al. 2015).

Third, research is needed to incorporate policy and other decision factors into retrofit design. This requires determining the appropriate weight for each item to enable condition-based optimization and prioritization (Tornaghi et al. 2018; Calvi et al. 2016). Nevertheless, some studies have argued that BCA is not a comprehensive method due to the challenges in monetizing some decision variables, such as business interruption during retrofits, the impact of structural upgrades on architectural aesthetics, and the need for specialized labor and technical design expertise. Clemett et al. (2023) proposed a multicriteria decision-making approach as an alternative method for optimizing combined retrofits, which may be a promising direction for a more comprehensive BCA that incorporates energy upgrades and other co-benefits into an economic evaluation.

## Conclusions

This study provides a literature-based roadmap to support cost-effective building design and retrofit practices in the United States, and useful insights to improve the accuracy and reliability of BCA. In particular, this study identifies key gaps and new areas of research that are important to future development of BCA methods and tools. Although this study focuses on earthquake risk reduction, the approaches and principles can be extended to support other forms of building performance enhancements such as reducing risk to other types of natural hazards and impacts.

The following conclusions can be drawn from our review regarding the cost-effectiveness of earthquake mitigation measures. Note that these conclusions may not apply to all cases.

- The cost-effectiveness of enhanced building design depends on a number of variables, including structural type, seismic hazard level, design strategy, construction quality, design life, and discount rate.
- The cost-effectiveness of seismic retrofit depends on a number of variables, including structural type, seismic hazard level, building height, building condition, retrofit method, remaining life of the building, and discount rate.
- Even in cases where full retrofit (in compliance with modern codes for new construction) is advised for safety reasons, it may not be economical, regardless of building height, structural type, and seismicity level. In contrast, partial retrofit that strengthens buildings to certain points slightly lower than modern code requirements can achieve sufficient safety and greater net benefits.
- High building performance may incur high construction or retrofit costs and lead to low net benefits. However, performance requirements should not be compromised when maximizing the economic benefits of a design or retrofit strategy.

When BCR falls below 1, the following tactics can be employed to increase the cost-effectiveness of a mitigation strategy based on our review:

- Prioritizing building groups for upgrade. As discussed, the cost-effectiveness of enhanced building design and seismic retrofit is affected by site conditions and building characteristics. The benefits are more pronounced for buildings at high seismic zones or near earthquake faults (FEMA 2020a; Porter et al. 2006), or old, tall buildings with the most vulnerable structural systems (Anagnos et al. 2016). While mitigation costs may also increase with a building’s vulnerability level (Fung et al. 2020), the benefit increase generally outweighs the cost growth (Porter 2021; NIBS 2019).
- Determining the optimal level of improvement. When buildings are upgraded to a certain level, additional investment may not further improve building performance or further increase economic benefits (Paxton et al. 2017; Vitiello et al. 2017; Kappos and Dimitrakopoulos 2008). There could be an optimal level of improvement that maximizes the benefits obtained for the amount paid.
- Setting multiple performance objectives. Designing or retrofitting buildings to meet multiperformance requirements (e.g., improved energy efficiency and reduced environmental impacts) may bring greater benefits to owners, tenants, and communities and increase overall cost-effectiveness (Pohoryles et al. 2020; Belleri and Marini 2016).

Understanding how to design or retrofit structures for earthquakes in a cost-effective way will lead to more widespread adoption and consequently improved resilience and safety. However, it is important to acknowledge the fact that a BCA may not include all of the relevant criteria for decision-making and should only be used as a guiding tool.

### Data Availability Statement

No data, models, or code were generated or used during the study.

## Figures and Tables

**Table 1.** Benefit–cost analysis methods for adopting or exceeding requirements of seismic codes

Study	Strategy	Performance objective^a^	Benefit	Cost	Analysis period	Method and data source

NIBS (2019)	Designing for 2018 I-Codes or exceeding 2015 I-Codes, compared with 1990s codes	LS and above	Avoided property loss, deaths, and injuries, direct and indirect business interruption, search and rescue	Assuming a 1% increase in cost with a 50% increase in strength and stiffness (Porter 2016)	75 years (2019–2094)	Hazus software, modified Hazus tabulated vulnerability functions, RSMeans cost data
NAHB (2018)	Designing for 2018 I-Codes, compared with 2015 I-Codes	LS	Not assessed	Added construction cost	Initial costs	RSMeans cost data, Census data, Bureau of Labor Statistics data, distributors’ or retailers’ websites
Nikellis et al. (2019)	Designing for ASCE 7–16 and AISC 341–10, compared with the criteria lower than ASCE 7–16	LS	Avoided structural and nonstructural damage	Added construction cost	50 years (2019–2069)	OpenSees software, PBEE approach, costs data from consulting firms and other studies
NEHRP (2013)	Designing for 2012 IBC, compared with 1999 SBC	LS	Reduced repair costs, fatalities, injuries, probability of collapse	Added construction cost	Annualized benefits; initial costs	PBEE approach, PACT software
Ryu et al. (2010)	Designing for 2009 NEHRP provisions, 2006 IBC, or 2003 IBC, compared with 1999 SBC	LS	Avoided structural and nonstructural damage	Not assessed	Annualized benefits	Memphis urban and adjusted national hazard curves, Hazus data
FEMA (2020a)	Designing for 2000 I-Codes (equivalent to 1997 UBC), compared with 1994 UBC	LS	Avoided physical and contents damage	Not assessed	Annualized benefits	Hazus software, CoreLogic parcel database, Microsoft footprint data
Kutanis and Boru (2014)	Designing for IO, compared with LS specified by Turkish seismic code TSC-07	IO	Annual losses (assuming no losses when buildings are designed for IO)	Added construction cost	Initial costs	Probina Orion software, Turkish governmental unit cost document

Note: LS = life safety; IO = immediate occupancy; NIBS = National Institute of Building Sciences; NAHB = National Association of Home Builders; I-Codes = the international codes; IBC = International Building Code; SBC = Standard Building Code; UBC = Uniform Building Code; NEHRP = National Earthquake Hazard Reduction Program; and PBEE = Performance-Based Earthquake Engineering.

a The performance objective applies to a building (group) under the design earthquake if no further specification.

**Table 2.** Benefit–cost analysis methods for seismic retrofits

Study	Building type	Performance objective^a^	Benefit	Cost	Analysis period	Method and data source

Meade and Kulick (2007)	Hospitals	IO	Not assessed	Retrofit and reconstruction, medical furnishings and equipment, nonstructural elements (e.g., cladding and roof tiles, mechanical and electrical equipment, elevators, utility systems)	Initial costs	OSHPD database, ATC database
Ghesquiere et al. (2006)	Hospitals	IO	Reduced property loss, direct and indirect deaths and injuries at the hospital, lives saved in earthquake-affected regions	Up-front fees paid on the total loan amount, commitment fees paid on the portion of the loan that has not been used yet, and the actual amount borrowed from banks	30 years	Probabilistic risk model
DGS (2002)	Concrete tilt-up and non-wood-frame school buildings	LS, DC	Not assessed	Engineering evaluation costs, program administrative costs, structural and nonstructural retrofit costs	Initial costs	FEMA 310 seismic evaluation method; FEMA 156 cost estimation method
Haghpanah et al. (2017)	Reinforced concrete school buildings	IO, LS^b^	Reduced structural and nonstructural damage, downtime, and injury and death rates	Not assessed	The simulated 1994 Northridge earthquake ground motion	SAP2000 software, Hazus software
Carofilis et al. (2020)	Reinforced concrete, precast concrete, and URM school buildings	Above LS	Reduced structural and nonstructural damage, repair costs, time, and injury and death rates	Retrofit	100 years	PBEE approach, OpenSees software, PACT software
Hutt et al. (2016)	Steel buildings	LS	Reduced structural and nonstructural damage, downtime	Not assessed	A magnitude 7.2 earthquake on the San Andreas Fault	PBEE approach, PACT software, REDi downtime estimation method
Dong and Frangopol (2016)	Steel buildings	LS^b^	Reduced repair costs and time, fatality loss, carbon emissions	Not assessed	The simulated 1940 El Centro earthquake and the 1995 Kobe earthquake	PBEE approach, OpenSees software
Harrington and Liel (2021)	Reinforced concrete buildings	CP, LS, IO	Repair costs	Not assessed	Annualized benefits	PBEE approach, OpenSees software, SP3 software
Vitiello et al. (2017)	Reinforced concrete buildings	LS	Not assessed	Retrofits, repairs, casualties and injuries, consequences of building unavailability	50 years	PBEE approach, SAP 2000 software
Liel and Deierlein (2013)	Reinforced concrete buildings	LS	Reduced fatalities and repair costs	Retrofit	50 years	PBEE approach, OpenSees software, RSMeans cost data, FEMA 156 document
Kappos and Dimitrakopoulos (2008)	Reinforced concrete buildings	IO, DC, LS	Not assessed	Retrofit, building damage, contents loss, rental loss, relocation, income loss, and casualties	40 years	COBE06 software
ATC (2010a)	Wood-frame buildings	IO, DC, LS^b^	Reduced structure and content losses	Retrofit	A magnitude 7.2 earthquake on the San Andreas Fault	Hazus software
Porter et al. (2006)	Wood-frame buildings	IO, DC, LS	Reduced repair costs	Retrofit	30 years	ABV method
Paxton et al. (2017)	URM buildings	LS	Reduced structural and nonstructural damage, downtime, and casualties	Retrofit	25, 50, and 70 years	Hazus software, assessor’s data
Gibson et al. (2014)	URM buildings	LS	Reduced building and contents damage, displacement costs, and casualties	Retrofit	30, 50, and 70 years	Hazus software

Note: URM = unreinforced masonry; IO = immediate occupancy; DC = damage control; LS = life safety; CP = collapse prevention; PBEE = Performance-Based Earthquake Engineering; OSHPD = California’s Office of Statewide Health Planning and Development; ATC = Applied Technology Council; and ABV = assembly-based vulnerability.

a The performance objective applies to a building (group) under the design earthquake if no further specification.

b The performance objective applies to a building (group) under a specific earthquake scenario.

**Table 3.** Cost-effectiveness of seismic retrofit strategies for performance objectives above life safety

Building type	Retrofit strategy	Region	BCR > 1	Reference

Reinforced concrete	Base isolation	California	Yes	Haghpanah et al. (2017)
	Strengthening beam-column joints with CFRP strips	Italy	No	Carofilis et al. (2020)
	Using steel braces and CFRP strips	Italy	No	Carofilis et al. (2020)
Precast concrete	Strengthening beam-column joints with steel braces	Italy	No	Carofilis et al. (2020)
	Using steel dowels and viscous dampers	Italy	Yes	Carofilis et al. (2020)
Wood frame	Using steel cantilevered columns and greater shear walls at ground floor	San Francisco	For a magnitude 7.2 earthquake on the San Andreas Fault	ATC (2010a)
URM	Attaching CFRP strips to both sides of masonry piers and spandrels	Italy	Yes	Carofilis et al. (2020)
	Using CFRP strips and viscous dampers	Italy	No	Carofilis et al. (2020)

**Table 4.** Cost-effectiveness of seismic retrofit strategies for the life safety performance objective

Building type	Retrofit strategy	Region	BCR > 1	Reference

Steel moment frame	Adding elastic spine frame with steel bracing	San Francisco	Not assessed	Hutt et al. (2016)
	Using earthquake-resilient nonstructural components	San Francisco	Not assessed	Hutt et al. (2016)
	Base isolation	San Francisco	Not assessed	Hutt et al. (2016)
		Los Angeles	Not assessed	Dong and Frangopol (2016)
Reinforced concrete	Concrete jacketing	Los Angeles, CA	Yes	Liel and Deierlein (2013)
		Italy	In high seismicity zones	Vitiello et al. (2017)
	FRP jacketing	Los Angeles	No	Liel and Deierlein (2013)
		Italy	In moderate, low, and very low seismicity zones	Vitiello et al. (2017)
	Installing shear walls	Los Angeles	Yes	Liel and Deierlein (2013)
		Italy	In high seismicity zones	Vitiello et al. (2017)
	Base isolation	Italy	In high and moderate seismicity zones	Vitiello et al. (2017)
Wood frame	Installing steel moment frames at garage openings	California	For locations near faults and on soft soil	Porter et al. (2006)
		San Francisco	For a magnitude 7.2 earthquake on the San Andreas Fault	ATC (2010a)
	Adding structural sheathing to the center longitudinal wall at ground floor	California	For locations near faults and on soft soil	Porter et al. (2006)
		San Francisco	For a magnitude 7.2 earthquake on the San Andreas Fault	ATC (2010a)
URM	Parapet bracing	Victoria, Canada	Yes	Paxton et al. (2017)
	Partial retrofit (installing shear and tension anchors at the roof and floors in addition to parapet bracing)	Victoria, Canada Seattle	Yes No	Paxton et al. (2017) Gibson et al. (2014)
	Full retrofit (strengthening walls, diaphragm, and columns in addition to partial retrofit)	Victoria, Canada	No	Paxton et al. (2017)

**Table 5.** Benefit-cost-transfer matrix

Stakeholder group	Construction cost	Property loss	Direct business interruption	Indirect business interruption	Insurance	Death and injury

Developer	—	2%	—	—	4%	—
Title holder	50%	58%	—	—	86%	—
Lender	—	7%	—	—	10%	—
Tenant	50%	33%	100%	—	—	99%
Community	—	—	—	100%	—	1%

Source: Data from NIBS (2019).

Note: Construction cost means up-front cost, which is assumed borne by the owner (title holder), who passes at most 50% of the cost to the tenant. Property loss includes repair costs and contents damage, which is assumed to account for a third of the property value. Direct business interruption is assumed to be fully borne by the tenant, while indirect business interruption is borne by the community. Insurance covers repairs borne by lenders, developers, and owners and the distribution is based on an assumed building service life of 75 years. Costs to the community due to death and injury are based on the assumption that average life insurance coverage in the US is about $60,000 per person, which equals 0.6% of the amount a community is willing to pay to avoid a statistical fatality; the total cost to the community can be up to 1% after including the coverage of nonfatal injuries.

**Table 6.** Examples of potential direct and indirect losses avoided associated with three levels of building functional recovery performance in an earthquake

Performance target	Direct loss	Indirect loss

Life safety	Repair costs	Deaths and injuries
Reoccupancy	Repair time	Displacement Deterioration of mental health Loss of social cohesion
Functional recovery	Recovery time	Business interruption Supply chain disruption

Source: Data from Fung et al. (2022a).
